# Outpatient mental healthcare service use among women with migrant background in Norway: a national register study

**DOI:** 10.1186/s12913-019-4788-4

**Published:** 2019-12-09

**Authors:** Melanie Straiton, Karina Corbett, Anna-Clara Hollander, Lars Johan Hauge

**Affiliations:** 1 Division of Mental and Physical Health, Norwegian Institute of Public Health, Box 222, Skøyen, 0213 Oslo, PO Norway; 2 Department of Public Health Sciences, Public Health Epidemiology, 171 77 Stockholm, Sweden

**Keywords:** Women’s mental health, Migrant health, Mental health services, Mood disorders, Neurotic and stress disorders

## Abstract

**Background:**

Studies show that migrant women are at greater risk of common mental disorders than the majority population, yet underrepresented in healthcare services. This study investigates the use of outpatient mental healthcare services over a five-year period among migrant and descendant women compared to majority women in Norway.

**Methods:**

Using linked national registry data, we selected all women resident in Norway between 2009 and 2013 (*N* = 1,834,822). We conducted generalised estimated equations with logistic regression to assess if the odds of using outpatient mental healthcare services differed for migrant and descendant women compared to majority women. We also conducted generalised estimated equations with negative binomial regression to calculate consultation incidence rate ratios for migrant and descendant women relative to majority women among those with a common mental disorder.

**Results:**

Both migrant and descendant women had lower odds (OR = 0.47 and OR = 0.60 respectively) of using outpatient mental healthcare services than majority women. Odds of using services increased with length of residency. We also found significant variation by country of origin. Among women with common mental disorders who had used services, migrants, but not descendants, had a lower consultation rate ratio than majority women. Analyses by region of origin revealed that this did not apply to women from EU European countries, North America and Australia and New Zealand.

**Conclusion:**

Women with migrant background are, overall, underrepresented in OPMH services. Findings indicate that migrant women may not only experience barriers to seeking and accessing care but also in maintaining access to care. This may especially be the case for newly arrived migrant women and women from non-Western countries. Treatment may not be culturally adapted for these groups. Closer investigation of the barriers migrant women experience after using OPMH services is required.

## Background

Migration is a risk factor for poor mental health, with both pre- and post-migration factors contributing to this risk [1, 2]. Studies suggest that migrants are at greater risk of common mental disorders such as mood disorders and neurotic and stress-related disorders (including anxiety and post-traumatic stress disorder) than the majority population [2–4]. This increased risk may also apply to descendants of migrants [5, 6].

Women are at greater risk than men of many common mental disorders [7]. The intersection of being both a woman and a migrant may therefore pose a double disadvantage. Migrant women experience more poverty, weaker attachment to the labour market, a greater childcare burden and are at greater risk of violence [8–10], all risk factors for mental disorders. Yet, research indicates that they are underrepresented in healthcare services for mental health difficulties [11–13]. We know even less about descendant women’s use of healthcare services. This paper investigates outpatient mental healthcare service utilisation in Norway among both migrant (foreign-born with foreign born parents) and descendent (Norwegian born with foreign born parents) women compared to majority women.

In contrast to research from North America where migrants tend to report better mental health than the majority population [14], European research suggests that many groups of migrants have poorer mental health [15–18]. However, there is considerable variation across different migrant groups [15]. Women from high income countries, for instance, tend to report better mental health than women from middle and low income countries [19]. Research on self-reported mental health among descendants often focuses on children and adolescents, and findings are relatively inconclusive [20]. In Europe, surveys of adult descendants tend to indicate that they report better mental health than their migrant counterparts but slightly poorer than the majority population [21, 22].

Despite reporting poorer mental health, migrant women are less likely to use healthcare services for mental health problems than majority women [13, 23]. Again, there is considerable variation in service use among different migrant groups and by length of residence, with those with longer residency being more likely to utilise services [13, 24]. Norwegian studies on healthcare service use often utilise register data but the extent to which differences are due to differences in mental health status or to barriers in seeking and accessing care is unclear. Difficulties in navigating the healthcare system, lack of information about available services, transport and language difficulties are well-documented barriers to initial access [25, 26]. Perceived stigma, lack of trust, different understandings of mental health, and thus perceived need for care, may also inhibit initial help seeking [26–28].

A Norwegian study among migrant women from Poland, Sweden, Russia, Pakistan, Thailand and The Philippines who had consulted GPs for mental health problems, found that those from countries outside of the European Union had lower odds of having conversation therapy with a doctor and were less likely to purchase psychotropic medicine than Norwegian women [29]. Thus, many migrant women appear to experience barriers beyond initial barriers for seeking and accessing care. Barriers to treatment or to maintaining care might include language incompatibility or communication difficulties with the healthcare provider, lack of culturally adapted services and providers’ failure to take account of differing understandings of mental health disorders [30, 31]. The latter can render the treatment solution seemingly less beneficial from the patient’s perspective.

Differences in treatment could alternatively be due to differences in mental health status. Indeed, it is possible that many women who consult GPs have fleeting or mild mental health difficulties due to a stressful period, which do not require additional follow-up or prolonged treatment. It is therefore of interest to know how service use varies among those with more severe mental health disorders requiring more follow up, such as among those who use outpatient mental healthcare (OPMH) services. An older, small scale Norwegian study looking at non-Western migrants found that women, in particular, were underrepresented in OPMH [12]. A national level study, from 2017, found large variation in mental healthcare service use by country of origin; those with migrant background from Iran and Iraq were more likely to use services compared with the majority population, while those with a background from Poland, Somalia, Sri-Lanka and Vietnam were less likely [24]. This study included both inpatient and outpatient care and considered contact with services at least once per year over four consecutive years. Total number of contacts were not reported. Although the study focused on people with migrant background, it did not discriminate between migrants and descendants of migrants, nor between men and women.

A study from the USA among adults with Somali background indicated that descendants reported lower levels of perceived stigma surrounding help-seeking for mental health disorders than migrants [32]. This may be because descendants are more exposed to the dominant culture than their parents are, and thus their understanding of, and attitude towards, mental health care may be more in line with that which is prevalent in the dominant culture. This increased exposure, as well as having been educated in the receiving country, means descendants will usually have superior language proficiency and a better understanding of how the healthcare system works than their migrant counterparts. Thus, descendants may experience fewer barriers to seeking, accessing and maintaining care compared to migrants.

To our knowledge, there are no large-scale European studies looking common mental health disorders, treated at the outpatient level, among women with migrant background that also discriminate between descendants and migrants. We also lack research on the rate of mental health service use among those who have *already* attended OPMH services. Lower repeated use may indicate barriers existing not just at the level of access but also in maintaining access to care. Using national registry data, we aim to look at the use of OPMH services among all women living in Norway between 2009 and 2013. We hypothesise that: 1) Migrant women will be less likely to have a least one OPMH consultation than majority women, but this will vary by length of residency and by region of origin. The difference between descendants and majority women will be smaller than for migrant women and majority women. 2) Migrant women with common mental disorders will have a lower OPMH consultation rate than majority women, but that this will vary by length of residency and region of origin. Descendants will not have a significantly different consultation rate to majority women.

## Method

### Study design, data sources and study population

This study was a national register-based cohort study, using a dynamic population. We used secondary data from three national registries/databases across a five-year period from 2009 to 2013: The Central Population Registry, The National Database for the Reimbursement of Health Expenses (KUHR) and The FD-Trygd database. Norwegian citizens, at birth, and all registered residents with a stay of six months or more are assigned a unique personal number. This personal number is used in a variety of contexts including tax and medical records. A de-identifiable version of this personal number, coded by Statistics Norway before issuing the data, was used to link the registries together at an individual level.

The Central Population Registry provides demographic data on all registered residents such as gender, year of birth and country of origin. Importantly, this data source also provides information on whether or not a person is residing in Norway in a given year. This is used to define study inclusion. The KUHR database includes reimbursement claims of fee-for-service made by healthcare practitioners in Norway. Among these, are claims from outpatient mental health services (OPMH). OPMH are localised services where both people with acute problems and those who need long-term follow-up can receive help. A referral from a doctor or psychologist is required. Each registration contains the date and type of contact. We consider all attended consultations, regardless of the type of contact. Diagnosis information from The International Statistical Classification of Diseases and Related Health Problems version 10 (ICD-10) is also recorded if a diagnosis is given. The FD-Trygd database is a historical event database and contains, among other information, data on welfare benefits and income.

All women residing in Norway at some point between 2009 and 2013, aged 16 to 67 years (identified using the Central Population Registry) were followed in order to investigate their OPMH service utilisation. Women were followed from the start of study period in 2009 or the start of the year they turned 16 years of age. Those who migrated after the start of the study period were followed from the year of migration. Censoring occurred at the end of the study period in 2013, the end of the year the women turned 67 (age of retirement) or the year of death or emigration, whichever came first. Migrants with less than one year residency were excluded if they were present during the study period for only that year. This was because those who moved in and out of the country within the same year were not likely to have had chance to use OPMH (e.g. it takes around 6 months to get a personal number and it is only after this that an individual is assigned a GP. A referral to OPMH services may also take some time).

### Study variables

Outcomes: At least one OPMH consultation in the study period (yes/no). For those diagnosed with a mood disorder (F30-F39 diagnosis in ICD-10) or a neurotic, stress-related or somatoform disorder (F40-F48 diagnosis) in OPMH services, the number of OPMH consultations during each year in study was the outcome.

Exposures: *Migrant category*: migrant (born outside of Norway with two non-Norwegian born parents), descendant (born in Norway, with two non-Norwegian born parents) and majority (all other women, including Norwegian born with at least one Norwegian parent and foreign born with at least one Norwegian parent). For migrants only we also considered *length of residency* (calculated from last year in study and year of arrival), and *region of origin:* 1) the Nordics, 2) Western Europe, North America, Australia and New Zealand (over 80% were from Western Europe and thus we shorten this to Western Europe etc), 3) EU Eastern Europe, 4) non-EU Eastern Europe, 5) Middle East/ North Africa, 6) sub-Saharan Africa, 7) South Asia and 8) East/South East Asia. Women from other countries made up less than 5% of the migrant population and were excluded from all analyses on region of origin.

Control variables: age and household income (including paid employment and benefits) after tax for each year. Those with an income of 60% or less of the median income level (EU definition of social deprivation) in any given year were defined as having low income.

### Statistical analysis

First, we conducted chi-square analyses and one way ANOVAs to assess for overall differences in demographics by migrant category and region of origin for the total sample. In descriptive data, age and length of residency are reported for the final year each person appears in the dataset. Low income is shown for those who had a low income at least once during the study period, and those who consistently had a low income throughout the period they were in the study (including those with only one year in the study).

For the first hypothesis, we calculated crude and age-standardised percentages of women with at least one OPMH consultation by migrant category, length of residency and region of origin for descriptive analyses. Age-standardised percentages were calculated according to the average age distribution of women in the whole population during 2009–2013 [33]. We conducted chi-square analyses to check for overall difference across the exposure variables. To test our hypothesis, we conducted generalised estimated equations (GEE) with a logistic regression model to assess whether migrant and descendant women had lower odds of having at least one OPMH consultation than majority women. GEE is a population-level approach which takes account of the longitudinal properties of the data. It allows us to take account of people who move in or out of the dataset, by including them only in analyses for the years they are present. Since age and income level varies considerable across the exposure variables, we controlled for age and income level for each year participants were in the study. We conducted separate analyses for majority women and migrant women with different lengths of residency and majority women and migrant women from different regions of origin. It was not possible to control for both in analyses with the majority population since length of stay is not a relevant variable. Thus, we also ran analyses with migrants only, where we could control for both region of origin and length of residency. South Asian women were set as the reference group since they had the longest length of residency.

For the second hypothesis, we selected out women who were diagnosed with a mood (affective) disorder (diagnosis codes: F30-F39) between 2009 and 2013 and considered the consultation rate over the five-year period. For our descriptive data analyses, we calculated the mean annual number of consultations (total number of consultations/years in study) and conducted Welch’s ANOVA to assess for overall differences across the exposure variables. To test our hypothesis, we estimated the consultation incidence rate ratios (IRRs) for migrant and descendant women compared to majority women by conducting GEEs with negative binomial regression, since our outcome (number of consultations) was over-dispersed count data. Again GEE analyses allowed us to account for people who moved in and out of the dataset by including them for the years in which they were present. Thus, this outcome measure is different from the mean annual number of consultations. We controlled for age and income level and conducted separate analyses for the majority population and migrants with different lengths of residency and from different regions. As before, we also carried out analyses on migrants only by region of origin and also controlled for length of residency. Finally, we repeated these analyses for women who were diagnosed with a neurotic, stress–related and somatoform disorder (diagnosis codes F40-F48) between 2009 and 2013.

## Results

### The study population

After excluding residents with less than one year residency in Norway who were present only for the same year (*N* = 26,993), our study population for hypothesis 1 (migrants will be less likely to use OPMH services than majority women) included 1,834,822 women; 82% were in the study for the full five-year period (mean = 4.57 years, (Sd = 1.03)). In analyses by region of origin, we excluded 12,824 migrant women from ‘other’ countries. Among those with an OPMH consultation (*n* = 173,592), 33% (*n* = 57,376) were diagnosed with a mood disorder (F30-F39) and 32% (*n* = 55,891) with a neurotic, stress-related or somatoform disorder (F40-F48). These women were included in separate analyses for hypothesis 2 (migrant women with a common mental health disorder will have a lower consultation rate ratio than majority women but descendants will not).

Overall, migrants made up around 15% of the population and descendants 1% percent. There was a significant age difference by migrant category (*F* (2)= 26,339.40, *p* < 0.001). Table 1 shows that migrants and especially descendants were younger than the majority. Household income level also differed substantially by migrant category (at least once in study period: *X*^2^ (2)=232,397.40, *p* < 0.001; throughout time in study: *X*^2^ (2)=176,963.07, *p* < 0.001).

**Table 1:** Demographics of women in study by migrant status and region of origin

	Majority women	Descendant women	All	Nordics	Western- Europe (etc)	EU Eastern Europe	Non-EU Eastern Europe	Middle East / North Africa	Rest of Africa	South Asia	East/ South East Asia
Total (*N*)	1,539,005	17,460	278,357	37,403	32,862	48,385	27,211	27,309	25,208	22,011	45,144
%	83.88%	0.95%	15.17%	2.04%	1.79%	2.64%	1.48%	1.49%	1.37%	1.20%	2.46%
% of migrants	–	–	–	14.09%	12.38%	18.22%	10.25%	10.28%	9.49%	8.29%	17.00%
Age (last year in study)											
Mean (sd)	43.33 (15.71)	24.89 (8.86)	37.95 (12.52)	39.88 (14.59)	40.85 (13.86)	35.42 (10.70)	38.79 (12.61)	37.23 (12.10)	34.24 (10.84)	39.20 (12.68)	37.92 (11.53)
Median	44	22	36	38	39	33	37	36	33	38	36
Low income at least once	320,500	7394	181,221	21,667	19,871	36,556	14,542	18,921	20,272	13,556	28,304
	20.83%	42.35%	65.10%	57.93%	60.47%	75.55%	53.44%	69.28%	80.42%	61.59%	62.70%
Low income throughout period in study	63,893	2052	75,897	10,016	9413	14,879	4703	7145	9610	5011	12,685
	4.15%	11.75%	27.27%	26.78%	28.64%	30.75%	17.28%	26.16%	38.12%	22.77%	28.10%
Length of residency (last year in study)											
1–2 years	–	–	60,055	9072	7520	15,333	3445	2591	5419	2839	11,469
			21.57%	24.25%	22.88%	31.69%	12.66%	9.49%	21.50%	12.90%	25.41%
3–5 years	–	–	53,970	5558	6967	16,837	3336	3718	5066	2621	7775
			19.39%	14.86%	21.20%	34.80%	12.26%	13.61%	20.10%	11.91%	17.22%
6–10 years	–	–	50,803	4468	5751	8253	6011	4951	6083	3484	9118
			18.25%	11.95%	17.50%	17.06%	22.09%	18.13%	24.13%	15.83%	20.20%
11–15 years	–	–	38,920	4682	3455	3150	5585	7102	4521	3534	5081
			13.98%	12.52%	10.51%	6.51%	20.52%	26.01%	17.93%	16.06%	11.26%
16–20 years	–	–	23,850	3536	1870	1473	6570	2827	1757	2249	2818
			8.57%	9.45%	5.69%	3.04%	24.14%	10.35%	6.97%	10.22%	6.24%
21+ years	–	–	50,759	10,087	7299	3339	2264	6120	2362	7284	8883
			18.24%	26.97%	22.21%	6.90%	8.32%	22.41%	9.37%	33.09%	19.68%

Table 1 also shows that among migrants, there was significant variation in age (*F* (8)= 1061,18, *p* < 0.001), income level (at least once in study period: *X*^2^ (8)=8389.15, *p* < 0.001, throughout time in study: *X*^2^ (8)=3898.30, *p* < 0.001) and length of residency (*X*^2^ (34)= 44,569.39, *p* < 0.001) by region of origin. The youngest groups were from EU Eastern Europe and sub-Saharan Africa, while the oldest groups were from Western Europe (etc), the Nordics and South Asia. Migrants from non-EU Eastern Europe were the least likely and migrants from sub-Saharan Africa were the most likely to have had both a low income at least once in the study period and throughout their time in the study.

Residency of over 20 years was most common among migrants from South Asia, while residency of less than 6 years was most common among EU Eastern Europeans.

### Hypothesis 1: at least one OPMH consultation

Table 2 shows the crude and age-standardised percentage of women attending OPMH services between 2009 and 2013 by migrant category and length of residency. Crude percentages were significantly different across the groups (*X*^2^ (2)=3614.95,*p* < 0.001). A lower percentage of migrant women than descendant and majority women had had a consultation. However, in GEE analyses with logistic regression (Table 2), both migrant and descendant women had far lower odds of using OPMH services than majority women, after controlling for age and income level.

**Table 2:** GEE^1^ Odds ratio and confidence intervals for OPMH consultation by migrant category and residency

	*N* with 1+ consultation	Crude%	Age-standardised%	GEE OR (95% CI)^2^
Majority	154,103	10.01%	10.64%	1.00^3^
Descendants	1703	9.75%	10.63%	0.60 (0.56–0.63)
Migrants	17,786	6.39%	6.52%	0.47 (0.47–0.48)
Majority				1.00^4^
0–2 years	975	1.62%	1.51%	0.16 (0.15–0.16)
3–5 years	2229	4.13%	4.12%	0.36 (0.34–0.37)
6–10 years	3655	7.19%	7.33%	0.56 (0.55–0.58)
11–15 years	3916	10.06%	9.90%	0.73 (0.71–0.76)
16–20 years	2478	10.39%	10.40%	0.89 (0.85–0.93)
21+ years	4533	8.93%	10.73%	1.05 (1.02–1.09)

^1^Generalised estimated equations; ^2^adjusted for age and income level ^3^based on 1,834,822 individuals (present in dataset on average 4.6 years), ^4^descendants excluded

Table 2 also shows a gradual increase in the percentage of women using OPMH services with increasing residency. Differences were significant (*X*^2^ (6)=7008.52, *p* < 0.001). After controlling for age and income level in GEE analyses with logistic regression, all migrants with less than 21 years residency had lower odds, while migrants with 21 or more years of residency had slightly higher odds of using OPMH services than the majority population.

OPMH consultations also varied by region of origin ((*X*^2^ (8)=6361.67, *p* < 0.001). Crude and age-standardised percentages of women using OPMH services by region of origin are shown in Table 3, together with results from GEE analyses with logistic regression. After adjusting for age and income, all groups of migrant women had lower odds an OPMH consultation than majority women. Compared with majority women, women from EU Eastern Europe and East/South East Asia had the lowest odds, while women from the Middle East/North Africa had the highest.

**Table 3:** GEE^1^ Odds ratio and 95% confidence intervals for OPMH consultation by region of origin

	*N* with 1+ consultation	Crude %	Age-standardised%	GEE OR (CI)^2^	GEE OR (CI)^2^	GEE OR (CI)^3^
Majority	154,103	10.01%	10.64%	1.00^4^		
Nordics	2499	6.68%	6.70%	0.53 (0.51–0.56)	0.96 (0.90–1.03)	1.00 (0.93–1.07)
Western- Europe (etc)	1775	5.40%	5.36%	0.44 (0.42–0.47)	0.77 (0.72–0.84)	0.87 (0.80–0.94)
EU Eastern Europe	1621	3.35%	3.90%	0.22 (0.21–0.24)	0.47 (0.43–0.50)	0.59 (0.55–0.65)
Non-EU Eastern Europe	2434	8.94%	9.12%	0.69 (0.66–0.72)	1.18 (1.10–1.27)	1.34 (1.25–1.44)
Middle East / North Africa	3497	12.81%	12.67%	0.94 (0.91–0.98)	1.83 (1.71–1.96)	1.91 (1.79–2.05)
Sub-Saharan Africa	1530	6.07%	6.20%	0.39 (0.67–0.41)	0.86 (0.79–0.93)	0.98 (0.90–1.07)
South Asia	1631	7.41%	7.34%	0.56 (0.53–0.59)	1.00^5^	1.00^5^
East/ South East Asia	1546	3.42%	3.59%	0.24 (0.22–0.25)	0.43 (0.40–0.47)	0.50 (0.46–0.54)

^1^Generalised estimated equations; ^2^adjusted for age and income level; ^3^ adjusted for age, income level and length of residency; ^4^based on 1,804,538 individuals (present in dataset on average 4.6 years); ^5^based on migrants only (present in dataset on average 4.2 years)

Analyses for migrants only are shown both before and after controlling for length of residency. Length of residency explained some, but not all, of the differences between different regions. Even after accounting for age, income level and length of residency, women from non-EU Eastern Europe and the Middle East had higher odds of using OPMH services while those from Western-Europe (etc), EU Eastern Europe and East/South East Asia had lower odds than women from South Asia.

### Hypothesis 2: OPMH consultation rate among those with common mental health disorders

The mean annual consultation rate was 3.32 (sd = 4.59) for women diagnosed with a mood disorder (F30-F39) and 3.03 (ds = 4.43) for women diagnosed with a neurotic, stress-related or somatoform disorder (F40-F48). Welsh’s ANOVA indicated a significant difference across migrant category for both F30-F39 disorders (*F* (2)=307.32, *p* < 0.001) and F40-F48 disorders (*F* (2)=246.03, *p* < 0.001). Table 4 shows that among women with F30-F39 disorders, majority women had the highest mean annual consultation rate while migrants had the lowest. GEE analyses with negative binomial regression showed that the consultation IRR was only slightly, but significantly lower, among migrant women compared with majority women after controlling for age and income level. Descendant women did not have a lower IRR than majority women.

**Table 4:** GEE^1^ Incidence Rate Ratios by migrant category and length of residency (common mental health disorders)

	Mood disorders			Neurotic, stress-related and somatoform disorders		
	*N*	Mean (sd) annual consultations	GEE IRR (95% CI)^2^	*N*	Mean (sd) annual consultations	GEE IRR (95% CI)^2^
Majority	50,334	3.37 (4.67)	1.00^3^	49,140	3.05 (4.52)	1.00^4^
Descendants	512	3.12 (4.05)	0.97 (0.95–1.00)	489	3.05 (3.80)	0.99 (0.97–1.02)
Migrants	6530	2.96 (3.95)	0.97 (0.96–0.98)	6262	2.81 (3.75)	0.98 (0.97–0.99)
Majority			1.00^5^			1.00^5^
0–2 years	259	3.16 (3.36)	0.86 (0.83–0.89)	280	3.62 (4.84)	0.83 (0.75–0.90)
3–5 years	719	2.61 (3.44)	0.95 (0.93–0.97)	739	2.62 (3.60)	1.01 (0.94–1.09)
6–10 years	1277	2.61 (3.47)	0.96 (0.94–0.98)	1312	2.58 (3.43)	0.94 (0.89–1.00)
11–15 years	1434	2.99 (4.01)	0.99 (0.97–1.00)	1459	2.82 (3.78)	0.92 (0.86–0.99)
16–20 years	966	3.13 (4.40)	0.98 (0.96–1.00)	921	2.91 (3.61)	0.98 (0.91–1.06)
21+ years	1875	3.19 (4.18)	1.00 (0.98–1.01)	1551	2.89 (3.90)	1.00 (0.94–1.07)

^1^Generalised estimated equations; ^2^adjusted for age and income level; ^3^based on 57.376 individuals (present in dataset on average 4.8 years); ^4^based on 55,891 individuals (present in dataset on average 4.8 years); ^5^descendants excluded

Among women with F40-F48 disorders, majority and descendants had the same mean annual number of consultations while migrants had fewer (Table 4). GEE negative binomial regression analyses confirmed that the consultation IRR did not significantly differ for descendants but was lower for migrants than for the majority population, even after controlling for age and income level.

Welch’s ANOVA also indicated that the annual mean number of OPMH consultations differed by length of residency for both F30-F39 (*F* (6)=800.74, *p* < 0.001) and F40-F48 disorders (*F* (6)=396.75, *p* < 0.001), as shown in Table 4. Among those with an F30-F39 disorder, the mean number of consultations was lowest for those with 3–5 and 6–10 years in Norway, and highest for those with 21+ years in Norway. After controlling for age and income in GEE analyses, all migrant women who had 10 or fewer years of residency had a lower IRR than majority women, while those with over 10 years residency did not significantly differ from the majority population.

Among those with an F40-F48 disorder, GEE analyses showed that only migrants with less than 2 years and between 11 and 15 years of residency had a lower IRR than the majority population, after controlling for age and income level.

By region of origin, we also saw a significant difference in mean annual number of consultations by region according to Welch’s ANOVA for both F30-F39 disorders (*F* (8)=232.12, *p* < 0.001) and F40-F48 disorders (*F* (8)=249.14,*p* < 0.001). Table 5 shows that among women with an F30-F39 disorder, the mean was highest among migrants from Western Europe (etc) and lowest amongst those from sub-Saharan Africa. After controlling for age and income level in GEE negative binomial analyses, all groups except for women from the Nordics, Western Europe (etc) and EU Eastern Europe had a significantly lower IRR than majority women. When comparing migrant women only, we found that Nordic and Western European (etc) women had a higher IRR than South Asian women but there were no differences for other groups. Length of residency made little difference to the IRR estimates.

**Table 5:** GEE^1^ Incidence Rate Ratios by region of origin (common mental health disorders)

	Mood disorders					Neurotic, stress-related and somatoform disorders				
	*N*	Mean (sd) annual consultations	GEE IRR (95% CI)^2^	GEE IRR (95% CI)^2^	GEE IRR (95% CI)^3^	*N*	Mean (sd) annual consultations	GEE IRR (95% CI)^2^	GEE IRR (95% CI)^2^	GEE IRR (95% CI)^3^
Majority	50,334	3.37 (4.67)	1.00^4^			49,140	3.05 (4.52)	1.00^5^		
Nordics	877	3.44 (4.17)	1.00 (0.98–1.02)	1.04 (1.01–1.08)	1.05 (1.01–1.08)	748	2.83 (3.95)	0.98 (0.95–1.01)	0.98 (0.95–1.02)	0.98 (0.95–1.02)
Western- Europe (etc)	714	3.64 (5.19)	1.02 (0.99–1.04)	1.06 (1.02–1.10)	1.07 (1.03–1.12)	528	3.04 (3.81)	1.01 (0.98–1.03)	1.01 (0.97–1.04)	1.01 (0.97–1.05)
EU Eastern Europe	570	3.11 (4.01)	0.98 (0.96–1.01)	1.02 (0.99–1.07)	1.04 (0.99–1.08)	515	2.89 (4.16)	0.98 (0.95–1.02)	0.99 (0.95–1.03)	0.99 (0.95–1.03)
Non-EU Eastern Europe	904	2.67 (3.50)	0.95 (0.92–0.67)	0.98 (0.95–1.02)	0.99 (0.96–1.03)	991	2.66 (3.31)	0.97 (0.95–0.99)	0.97 (0.94–1.00)	0.97 (0.94–1.01)
Middle East / North Africa	1438	2.74 (3.38)	0.95 (0.94–0.97)	0.99 (0.96–1.03)	0.99 (0.96–1.03)	1476	2.79 (3.47)	0.98 (0.96–0.99)	0.98 (0.95–1.02)	0.99 (0.95–1.02)
Sub-Saharan Africa	420	2.52 (3.96)	0.92 (0.88–0.95)	0.96 (0.91–1.01)	0.97 (0.92–1.02)	529	2.68 (3.99)	0.95 (0.92–0.98)	0.97 (0.93–1.01)	0.97 (0.93–1.01)
South Asia	585	2.81 (3.63)	0.96 (0.93–0.98)	1.00^6^		643	2.96 (3.97)	0.99 (0.97–1.02)	1.00^6^	1.00^6^
East/ South East Asia	532	2.72 (3.90)	0.95 (0.92–0.98)	0.99 (0.95–1.03)	0.99 (0.95–1.04)	422	2.54 (3.69)	0.96 (0.92–0.99)	0.96 (0.91–1.00)	0.96 (0.91–1.00)

^1^ Generalised estimated equations ^2^adjusted for age and income level; ^3^ adjusted for age, income level and length of residency; ^4^analysis based on 56.374 individuals (present in dataset on average 4.8 years); ^5^based on 54, 992 individuals (present on average 4.8 years); ^6^based on migrants only (present on average 4.8 years)

For women with an F40-F48 disorder, those from Western Europe (etc) also had the highest mean number of annual consultations, while women from East/South East Asia had the lowest. After controlling for age and income level, women from non-EU Eastern Europe, Middle East, North Africa, sub-Saharan Africa and East/South East Asia had a lower IRR than majority women. When comparing only migrant groups, however, we found no significant differences between South Asian women and women from other regions. Including length of residency made no difference to the IRR estimates.

## Discussion

In this study, we aimed to gain more insight into migrant and descendant women’s use of OPMH services compared with majority women using national level registry data. Overall, our results suggest that migrant and descendant women use OPMH services to a lesser extent than majority women. Descendant women are less likely to use OPMH services, while migrant women are both less likely to use OPMH services and have fewer follow-up consultations for common mental health disorders. However, there are substantial differences by both length of residency and region of origin. Previous Norwegian studies on mental health service use have either focused on primary care, been based on small samples, not considered the number of consultations over an extended period or not mentioned migrant *women* or descendants in particular [13, 24, 34]. Thus, this paper fills an important gap.

As expected, the difference in odds ratios between migrants and the majority population attending OPMH services decreased with increasing length of residency. Women with more than 20 years of residency had slightly higher odds while all other groups had lower odds compared with the majority population. Further, controlling for length of residency among migrant women appeared to account for some of the difference in odds of consultations. This could lend support to the ‘exhausted migrant effect’, where health declines as a result of poor living and working conditions [33]. Self-report studies show that migrants from high income countries tend to report better mental health than other migrants [19]. Women from African countries such as Somalia also report a relatively low level of mental distress compared with women from Middle Eastern countries [18, 35]. This is similar to research in other European countries [36, 37]. Thus, to some extent the differences in odds we observed between different groups of migrant women using OPMH may reflect actual differences in mental health; for example, the percentage of migrant women using OPMH services was highest amongst women from the Middle East/ North Africa. Yet, all groups had lower odds than majority women which would not be expected based on self-reported studies [18]. Therefore, consultation rates cannot only reflect underlying mental health differences.

There may also be substantial differences in access. Barriers to care may decrease steadily over time. A Portuguese qualitative study found that migrant women with longer residency tended to report fewer access difficulties compared with migrant women with shorter residency [38]. A Canadian study also suggested that migrants with longer residency had a better understanding of how to access and use health services [39]. Thus, it is possible that migrant women with longer residency in Norway are better equipped to seek and successfully navigate OPMH services than more newly arrived migrant women. Similarly, higher levels of stigma around mental health disorders may be prevalent among some migrant groups but lessen over time. A qualitative study among Filipina migrants in Norway suggested that women who had more information about mental health, or had contact with someone with a mental health disorder after migrating, endorsed less stigmatising attitudes [27].

Navigations barriers are likely to be less challenging for descendants, who have grown up with the Norwegian healthcare system. Similarly, stigma may be less prevalent than among their migrant counterparts [32]. We therefore expected that descendants’ use of OPMH services would be closer to that of the general population. Yet, after controlling for age and income level, majority women were over 66% more likely to use OPMH services than descendant women. It is unlikely that this reflects differences in mental health status, since in both Norwegian and international research, adult descendants’ mental health status suggests that descendants do not have a mental health advantage over the majority population [21, 40]. In line with our finding, a Danish study also found that descendants with two-foreign born parents were less likely to have a psychiatric consultation than the majority population [5]. Therefore, it is possible that descendants they actually experience greater barriers to seeking and accessing mental health care than the majority population. Indeed, a study among Pakistanis in Germany found that descendants faced similar barriers to accessing the healthcare system as migrants [41]. We need further research that can address actual mental health status and healthcare needs among descendants, as well as investigate the barriers they face.

By selecting out women who had the same type of diagnosis (both mood disorders and neurotic or stress-related disorders) and looking at their rate of service use between 2009 and 2013, we were able to reduce differences in both mental health status and initial barriers in accessing OPMH services. Thus, remaining differences may indicate barriers in maintaining access to care. Descendant women did not have a lower consultation rate ratio than majority women, thus, confirming our hypotheses. This indicates that descendant women do not appear to experience much inequity in care or barriers after initially accessing services compared with majority women. Among migrant women, our hypotheses were also supported. Overall, migrant women with a mood disorder had a lower IRR than majority women, as expected, and women with less than 11 years of residency had a lower consultation rate ratio than majority women. This may suggest that migrant women can experience barriers in maintaining care such as language differences, differing understandings of mental health disorders, cultural suitability of care or experiences of discrimination. While out of pocket consultation fees are relatively small and capped at around 240 euros per year, the cost could still be an obstacle for migrants on lower incomes [27]. Some of the above barriers may decrease with length of residency.

Yet, surprisingly, we found that among women with a neurotic or stress disorder, only those with a length of residency of 0–2 years and 11–15 years had a lower IRR than majority women after accounting for age and income level. It is possible that length of residency relates to region of origin and that region of origin is more important in explaining consultation rates than length of stay. Indeed, adjusting for length of residency made little difference to the IRR estimates by region of origin among migrants. Among women with a mood disorder, we found that women from non-EU Eastern Europe, the Middle East/North Africa, sub-Saharan Africa, South Asia and East/South East Asia had a lower consultation rate ratio compared with majority women. Findings were similar for women with a neurotic/stress disorder, except South Asian women did not have a lower rate ratio than majority women. This confirmed our hypothesis that consultation rate ratios would vary by region of origin. Strikingly, it seems that women from countries outside of the EU, North America and Australia and New Zealand (i.e. countries often referred to as non-Western) may experience greater inequity in care. This is similar to findings in primary care [29]. Treatment may not be adapted to take account of different understandings of mental health disorders and/or preferences for care. Thus, these groups may perceive treatment as less relevant and be less likely to continue treatment.

On the other hand, we cannot rule out that variation in consultation rate ratios are due to differences in mental health status. Although all women had for instance, mood disorders, there are many different diagnoses within this, which may require a different treatment intensity. It could therefore be that, for instance, women with mood disorders and less than 11 years of residency, on average, have less complex mental disorders than the majority women, or that migrant women from non-Western countries recover faster from mood disorders than majority women. A recent Canadian study found that Chinese and South Asian minorities presenting at inpatient psychiatric hospitals had shorter stays and greater improvement in functionality at discharge compared with other patients [42]. There is a need for studies that provide more detailed information about mental health status among women with different migrant backgrounds at different points in the treatment trajectory.

Another limitation of this study is that we only considered one type of specialist mental healthcare use. We did not consider inpatient services, use of private psychologists and psychiatrists or other sources of help that may be relevant for migrants. However, inpatient services only make up around 5% of contacts with mental healthcare services [43]. Additionally, given that private services are more costly, migrants, who are more likely to experience social deprivation, may be less likely to use private services than the majority population. Further, we only considered consultation rates for mood and neurotic and stress-related disorders. Previous research indicates that migrants and descendants may also be at higher risk of psychotic disorders than the majority population [5, 44]. However, such diagnoses are less common and are more likely to be treated at inpatient service level. Additionally, the data used in the study are now several years old and the composition of migrant groups may be different. Finally, there are a number of confounders we did not control for including education, employment, geographical region, previous living conditions and migration-related experiences which are likely to impact both mental health status and use of OPMH services. Despite these limitations, the findings from this national level study offer insight into how the use of OPMH services varies by migrant status, country of origin and length of stay. Our study design allowed the inclusion of data from all women in Norway during the time period, while taking account of the number of years each contributed to the study. Thus, there is little selection bias.

## Conclusion

Women with migrant background are, overall, underrepresented in OPMH services. In order to tease apart the extent to which migrant and descendant women’s underrepresentation is due to better mental health or to greater barriers among different groups, we need more studies looking at the gap between mental health status and mental health service use. Nonetheless, our study indicates that women with migrant background do not only experience barriers to seeking and accessing care but may also experience barriers in maintaining access to care. This may especially be the case for newly arrived migrant women and women from non-Western countries. Closer investigation of the barriers migrant women experience after using OPMH services is required. This can help identify the ways in which services can adapt treatment for women from different regions of the world, both in Norway and elsewhere.

## Data Availability

The datasets generated and analysed for the current study are not publicly available due to data protection reasons. However, the data that support the findings of this study may be available from Statistics Norway and HELFO if ethical approval is granted.

## Abbreviations

CI: Confidence interval
EU: European Union
GEE: Generalised estimated eqs.
GP: General practitioner
ICD-10: International Statistical Classification of Diseases and Related Health Problems version 10
IRR: Incidence rate ratio
KUHR: The Control and Payment of Health Reimbursement Database
OPMH: Outpatient mental healthcare
OR: Odds ratio
Sd: Standard deviation
